# Comparative Analysis of Salt Tolerance and Transcriptomics in Two Varieties of *Agropyron desertorum* at Different Developmental Stages

**DOI:** 10.3390/genes16040367

**Published:** 2025-03-22

**Authors:** Yuchen Li, Xintian Huang, Xiao Han, Hui Yang, Yan Zhao

**Affiliations:** College of Gressland Science, Inner Mongolia Agricultural University, Hohhot 010018, China; liychen2024@126.com (Y.L.); huangxintian1998@126.com (X.H.); 15174926059@163.com (X.H.); nuohui@163.com (H.Y.)

**Keywords:** *Agropyron desertorum*, salt stress, RNA-seq, physiological analysis, seed germination, transcriptomics

## Abstract

Background: Most of the grasslands in China are experiencing varying degrees of degradation, desertification, and salinization (collectively referred to as the “three degradations”), posing a serious threat to the country’s ecological security. *Agropyron desertorum*, known for its wide distribution, strong adaptability, and resistance, is an excellent grass species for the ecological restoration of grasslands affected by the “three degradations”. This study focused on two currently popular varieties of *A. desertorum*, exploring their salt tolerance mechanisms and identifying candidate genes for salt and alkali tolerance. Methods: Transcriptome sequencing was performed on two varieties of *A. desertorum* during the seed germination and seedling stages under varying degrees of saline–alkali stress. At the seed stage, we measured the germination rate, relative germination rate, germination index, and salt injury rate under different NaCl concentrations. During the seedling stage, physiological indicators, including superoxide dismutase (SOD), peroxidase (POD), malondialdehyde (MDA), proline (PRO), soluble protein (SP), and catalase (CAT), were analyzed after exposure to 30, 60, 120, and 180 mM NaCl for 12 days. Analysis of differentially expressed genes (DEGs) at 6 and 24 h post-treatment with 120 mM NaCl revealed significant differences in the salt stress responses between the two cultivars. Results: Our study indicates that during the seed stage, *A. desertorum* (Schult.) exhibits a higher relative germination potential, relative germination rate, and relative germination index, along with a lower relative salt injury rate compared to *A. desertorum* cv. Nordan. Compared with *A. desertorum* cv. Nordan, *A. desertorum* (Schult.) has higher salt tolerance, which is related to its stronger antioxidant activity and different antioxidant-related pathways. Gene Ontology (GO) and Kyoto Encyclopedia of Genes and Genomes (KEGG) enrichment analyses were used to identify the key biological processes and pathways involved in salt tolerance, including plant hormone signal transduction, antioxidant defense, and cell membrane stability. Conclusions: *A. desertorum* (Schult.) exhibits stronger salt tolerance than *A. desertorum* cv. Nordan. Salt stress at a concentration of 30–60 mM promotes the germination of the seeds of both *Agropyron cultivars*. The two *Agropyron* plants mainly overcome the damage caused by salt stress through the AsA-GSH pathway. This study provides valuable insights into the molecular mechanisms of salt tolerance in *Agropyron* species and lays the groundwork for future breeding programs aimed at improving salt tolerance in desert grasses.

## 1. Introduction

Soil salinization is a type of abiotic stress that significantly restricts agricultural productivity, especially in arid and semi-arid regions. Salinization threatens the growth of plants, constituting 20% of arable land and nearly half of irrigated areas around the world unsuitable for producing food [1]. It results in the permanent loss of vegetation and significant decreases in both the yield and quality of food and forage, reducing arable land. Such impacts threaten agricultural and pastoral production systems and degrade ecological environments, restricting agricultural development and limiting improvements in living standards. Therefore, improving and using saline–alkali land have become critical challenges. This effort is essential not only for expanding arable land resources but also increasing green vegetation coverage and enhancing ecological stability [2]. Additionally, it plays a key role in comprehensive land management strategies. Using saline–alkali land for forage production, a means of merging the reclamation of saline–alkali land and animal husbandry, is one of the most basic methods for maximizing ecological and economic benefits [3,4]. Thus, the selective breeding of salt-tolerant plant species and understanding plant salt tolerance mechanisms are crucial for developing salt-resistant crops.

*A. desertorum* (Fisch. ex Link) Schult., commonly known as desert wheatgrass, is a perennial herbaceous plant belonging to the Poaceae family and the *Agropyron* genus. It thrives in arid grasslands, sandy soils, hills, and dunes and is well known for its adaptability to harsh environmental conditions, including salinity, drought, cold, and wind erosion [5,6,7]. This species is frequently used for soil stabilization, dune fixation, and saline–alkali land reclamation [8]. Similarly, *A. desertorum* (Fisch.) Schult. cv. Nordan, a cultivar of *A. desertorum*, commonly referred to as *A. desertorum* Nordan, also exhibits remarkable environmental adaptability. It flourishes in extremely arid and nutrient-poor northern regions, as well as in cold and water-deficient environments [9,10]. Due to its resilience, *A. desertorum* Nordan holds significant potential for saline–alkali land improvement and use [11].

Despite the known ecological importance of these two varieties, the molecular mechanisms underlying their salt tolerance, particularly during seed germination and seedling growth, remain poorly understood. De novo transcriptome analysis provides a powerful approach for understanding the genetic responses of non-model species under stress conditions, such as salinity. By examining gene expression profiles under NaCl stress, it is possible to identify key regulatory pathways and genes involved in salt tolerance mechanisms.

The osmotic adjustment capacity of cells enables plants to withstand certain environmental stresses. Osmotic adjustment involves the active accumulation of hydrophilic small molecules by cells to prevent passive dehydration. In response to osmotic stresses such as salinity, plants actively accumulate osmotic regulators, including inorganic ions (Ca^2+^, K^+^, Cl^−^) and organic solutes (proline, soluble proteins, betaine, glycine, trehalose, sucrose, and other small organic molecules) [12]. Within the plant antioxidant defense system, both enzymatic and non-enzymatic components detoxify and scavenge reactive oxygen species (ROS), mitigating their harmful effects [13]. The ascorbate–glutathione (AsA-GSH) pathway encompasses many of these enzymatic and non-enzymatic antioxidants and serves as a crucial defense mechanism under salt stress. It is also a key indicator of a plant’s capacity to resist oxidative and osmotic stress [14]. Ascorbate (AsA) and glutathione (GSH) are present in all cellular compartments and cell types, aiding in mitigating oxidative-stress-induced damage and maintaining cellular homeostasis. As the primary intracellular antioxidants, AsA and GSH can interconvert to scavenge ROS, forming the AsA-GSH cycle to protect cells from oxidative damage [15]. In this cycle, enzymes such as ascorbate peroxidase (APX), glutathione S-transferase (GST), and glutathione peroxidase (GPX) play pivotal roles in detoxification. APX reduces H_2_O_2_ using AsA, maintaining antioxidant balance and enhancing plant tolerance to oxidative stress [16]. GST catalyzes the conjugation of GSH with harmful substances, forming more hydrophilic compounds that are more easily excreted, thereby reducing the accumulation of harmful substances within cells [17]. GPX uses GSH to catalyze the reduction in H_2_O_2_ and organic peroxides into water or corresponding alcohols, thereby decreasing the toxicity of these ROS, maintaining cellular redox balance, and preventing oxidative damage to cells [18]. In summary, the enzymatic and non-enzymatic antioxidants in the AsA-GSH pathway work synergistically to maintain ROS homeostasis within cells, protecting plant cells from oxidative stress and thereby enhancing plant survival under adverse conditions.

Seed germination is a critical phase for establishing stable seedlings, determining the crop population size [19]. In studies of gramineous plants, salt stress has been found to significantly inhibit the seed germination of *Oryza sativa* [20] and Triticum aestivum [21], resulting in stunted seedlings. Research on *Arabidopsis thaliana* has revealed that salt stress can cause the leaves to become smaller and inhibit root growth [22]. Regarding *A*. *desertorum*, salinity stress (NaCl) significantly affects its early phenological stages, while it exhibits some resistance during later developmental phases [23]. In screening germplasm resources of barley (*Hordeum vulgare*), wheat (*Triticum aestivum*), rice (*O. sativa*), and maize (*Zea mays*) for salt tolerance, researchers commonly use growth indicators such as germination vigor, germination rate, shoot length, and root length as evaluation criteria [24,25,26,27]. For assessing salt tolerance during the seedling stage, indicators such as antioxidant enzymes (e.g., SOD, POD, CAT), MDA content, chlorophyll content, electrical conductivity, soluble substances, and sodium/potassium (Na^+^/K^+^) ion concentration are commonly used as references [28,29].

In this study, we compared and analyzed the germination performance and physiological responses of *A. desertorum* Schult and *A. desertorum* Nordan under salt stress during both the seed and seedling growth stages. We also studied the transcriptomic differences between the two varieties of *A. desertorum* after salt stress. This comprehensive evaluation of *A. desertorum* Schult and *A. desertorum* Nordan’s salt tolerance aimed to identify key genes and pathways responsible for their differential responses. The findings provide valuable insights for breeding salt-tolerant and osmo-tolerant plant varieties suitable for saline and high-osmotic-stress environments.

## 2. Materials and Methods

### 2.1. Plant Materials and Treatment Conditions

**Figure 1:**
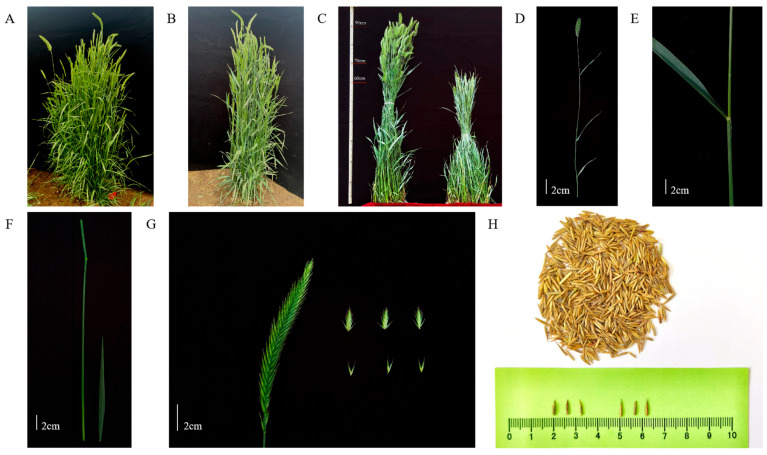
*A. desertorum* plant varieties. (**A**) *A. desertorum* (Fisch.) Schult single-plant picture. (**B**) *A. desertorum* (Fisch.) Schult. cv. Nordan single-plant picture. (**C**) Comparison of two varieties of *Agropyron*. (**D**) *A. desertorum* (Fisch.) Schult reproductive branch picture. (**E**) *A. desertorum* (Fisch.) Schult leaf picture. (**F**) *A. desertorum* (Fisch.) Schult stem picture. (**G**) *A. desertorum* (Fisch.) Schult flower spike, spikelet, and floret picture. (**H**) *A. desertorum* (Fisch.) Schult seed picture. Note: (**C**) The left side is *A. desertorum* (Fisch.) Schult and the right side is *A. desertorum* (Fisch.) Schult. cv. Nordan. (**G**) The left picture shows a spike, the top-right picture shows spikelets, and the bottom-right picture shows florets.

To accurately evaluate the performance of two varieties of *Agropyron* under sodium chloride treatment, this study conducted a comparative analysis of the relevant index parameters of *A. desertorum* (Fisch.) Schult (Figure 1A) and *A. desertorum* (Fisch.) Schult. cv. Nordan (Figure 1B) under salt stress. Under normal growth conditions, the biomass and plant height of *A. desertorum* (Fisch.) Schult are higher than those of *A. desertorum* (Fisch.) Schult. cv. Nordan (Figure 1C). The original sources of the plant materials were introduced from the germplasm bank of Inner Mongolia Agricultural University (Figure 1D,H).

#### 2.1.1. Seed Stage

**Figure 2:**
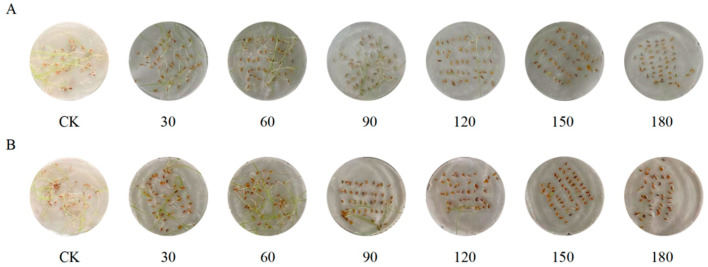
Pictures of the seedlings of two varieties of *A. desertorum*. (**A**) Pictures of *A. desertorum* (Fisch.) Schult. (**B**) Pictures of *A. desertorum* (Fisch.) Schult. cv. Nordan. Note: The NACL stress concentration was measured in mM.

The plant materials used in this study, *A. desertorum* Schult and *A. desertorum* Nordan, are varieties of *A. desertorum* (Fisch. ex Link) Schult. These varieties were introduced and bred by Professors Jinfeng Yun and Yan Zhao at Inner Mongolia Agricultural University and are preserved in the university’s seed bank. Both the seed and seedling experiments were conducted at the Key Laboratory of Grass Science at Inner Mongolia Agricultural University. Mature, plump and uniformly sized seeds of the two varieties were selected for stress treatment during the seed stage. The seeds were disinfected with a 0.1% mercuric chloride solution for 20 min. The seeds were then placed evenly in Petri dishes (9 cm in diameter) lined with double layers of sterile filter paper. For each Petri dish, 8 mL of NaCl solution was added at concentrations of 0.0 mM (ddH_2_O), 30 mM, 60 mM, 90 mM, 120 mM, 150 mM, and 180 mM (Figure 2). The seeds were incubated under controlled conditions of 25 °C with a 14 h light/10 h dark photoperiod. Three dishes were used per treatment, and each treatment was replicated three times.

During seed germination, the seeds were observed, and the germination status was recorded every three days until no new shoots emerged. On day 7, the germination potential of the seeds was recorded. The seed germination rate (indices of seed quality and germination ability), relative germination rate (indices of germination characteristics under different treatments), germination potential (indices for measuring seed germination speed and uniformity), relative germination potential (indices for measuring the germination characteristics of seeds under different treatments), germination index (reflects the dynamic process of seed germination), and relative germination index (measures the relative germination ability and characteristics of seeds under specific treatments) were recorded to calculate the relative salt injury rate and salt tolerance indices of the two plant materials. This comprehensive approach provided a thorough evaluation of the salt tolerance of the two varieties in the seed stage.

#### 2.1.2. Seedling Stage

After completing the data collection during the seed stage, uniform seedlings that had reached the three-leaf stage were selected from the control group and transplanted into a hydroponic device for stress treatment. Twelve days after the stress treatment, the seedling phenotypes were recorded and physiological indicators were measured. Phenotypic indicators included leaf length, leaf width, and root length. Physiological indicators included superoxide SOD, POD, PRO, SP, MDA, and CAT activity or content.

#### 2.1.3. Plant Treatments for Transcriptome Sequencing

*A. desertorum* (Schult.) and *A. desertorum* cv. Nordan plants at the three-leaf stage were selected for the transcriptome sequencing analysis. The experimental group was subjected to salt treatment with a 120 mM sodium chloride solution, while the control group was treated with distilled water. Sampling was conducted with three biological replicates and three technical replicates at 0 h, 6 h, and 24 h after the treatment. The samples of *A. desertorum* (Schult.) were labeled as S_CK (S120_0h), S_6 (S120_6h), and S_24 (S120_24h) at 0 h, 6 h, and 24 h after the salt treatment, respectively. The samples of *A. desertorum* cv. Nordan were labeled as N_CK (N120_0h), N_6 (N120_6h), and N_24 (N120_24h) at 0 h, 6 h, and 24 h. Whole seedlings were sampled, and transcriptome sequencing was conducted.

### 2.2. RNA Extraction and Sequencing

Total RNA was extracted from whole seedlings of *A. desertorum* Schult and *A. desertorum* Nordan treated with 0 mM and 120 mM sodium chloride (NaCl) at 0 h, 6 h, and 24 h post-treatment using the TRIzol (Thermo Fisher Scientific, Waltham, MA, USA) reagent. The quality of RNA was evaluated using the Agilent 2100 Bioanalyzer (Agilent, Technologies, Palo Alto, CA, USA). The NEBNext^®^ Ultra™ RNA Library Prep Kit (Thermo Fisher Scientific, Waltham, MA, USA) was used to construct RNA sequencing libraries. High-throughput sequencing was carried out on the Illumina HiSeq 2500 platform (Illumina, San Diego, CA, USA). The raw sequencing reads were subjected to quality inspection using FastQC (Babraham Bioinformatics, Cambridge, UK), and low-quality reads and adapter sequences were removed by Trimmomatic (USADeLLab, Gainesville, FL, USA). The high-quality (clean) sequencing reads were retained for further analysis.

### 2.3. Quality Control, Transcriptome Assembly, and Annotation of Unigenes

Raw reads generated from the Illumina HiSeq 2500 platform were subjected to quality control to remove adapter sequences, poly-N tails, and low-quality reads using FastQC and Trimmomatic. The clean data obtained after this filtering process were used for downstream analyses.

De novo transcriptome assembly was performed using Trinity software (version: 2.4, Beijing Novogene Company, Beijing, China) [30], following standard protocols as described in previous studies. This assembly resulted in a comprehensive set of unigenes.

Functional annotation of the assembled unigenes was conducted using the BLASTX algorithm (E-value < 1.0 × 10^−5^) to compare sequences against multiple databases, including the Nucleotide (Nt, https://www.ncbi.nlm.nih.gov/nucleotide/, accessed on 25 August 2022), Non-redundant protein (Nr, https://www.ncbi.nlm.nih.gov/protein/, accessed on 27 August 2022), Gene Ontology (GO, http://geneontology.org/, accessed on 29 August 2022), Pfam (http://pfam.xfam.org/, accessed on 30 August 2022), Swiss-Prot (https://www.uniprot.org/, accessed on 1 September 2022), COG (https://www.ncbi.nlm.nih.gov/COG/, accessed on 1 September 2022), KEGG (http://www.genome.jp/kegg/, accessed on 4 September 2022), and KEGG Orthology (KO, https://www.genome.jp/kegg/ko.html, accessed on 6 September 2022) databases. To ensure comprehensive functional insights, all unigenes were screened across these databases.

Using Nr annotation, the unigenes were further categorized into Gene Ontology (GO) functional groups, namely biological processes (BPs), molecular functions (MFs), and cellular components (CCs), employing Blast2GO online software (https://www.blast2go.com/, accessed on 1 September 2022). Additionally, the pathway distribution of unigenes was analyzed based on the KEGG database using the BLASTX algorithm to identify the metabolic and regulatory pathways involved in the salt stress response.

### 2.4. Differential Gene Expression Analysis

Clean reads obtained from RNA sequencing were aligned with the de novo-assembled transcriptome using Bowtie 2 (version: bowtie2-2.5.2) [31], and gene expression levels were quantified using RSEM (RNA-Seq by Expectation-Maximization) (version: 1.3.1). The expression levels of each gene were normalized to transcripts per million (TPM) to account for differences in sequencing depth and gene length [32].

DEGs between the NaCl-treated (6 h and 24 h) and control (0 h) samples were identified using the DESeq2 (version: 1.36.0) [33] package. Genes with a |log2 fold change| ≥ 2 and an adjusted *p*-value < 0.05 were considered significantly differentially expressed. The Benjamini–Hochberg method was applied to control the false discovery rate (FDR).

Functional enrichment analysis of DEGs was performed using the GO and KEGG databases. GO analysis categorized DEGs into three main functional groups: biological processes, molecular functions, and cellular components. All statistical analyses and visualizations of DEGs were conducted using R software (version: 4.2.1), and functional enrichment was performed using the clusterProfiler package (version: 4.5.2).

## 3. Results

### 3.1. Salt Treatment Performance at Seed Stage

**Figure 3:**
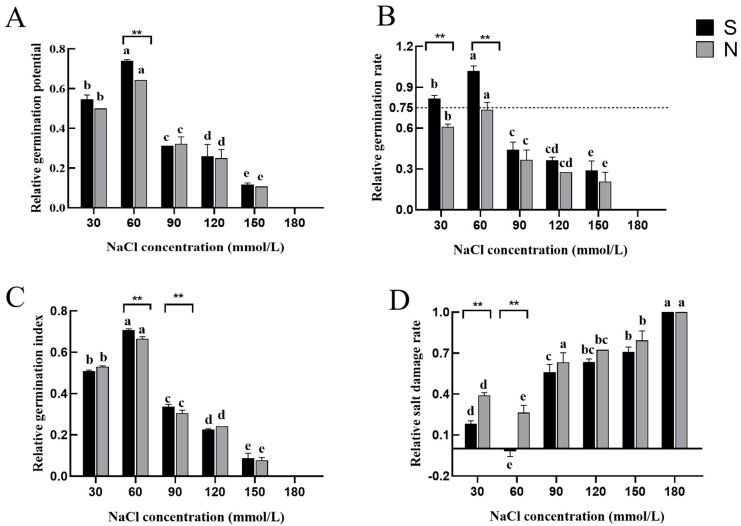
Measurement of seed-stage parameters in two varieties. (**A**) Relative germination potential on day 7 for both varieties. (**B**–**D**) Relative germination rate, relative germination index, and relative salt injury rate under salt stress, respectively. Note: S: *A. desertorum* Schult; N: *A. desertorum* Nordan. The dotted line is regarded as the suitable range of salt tolerance of seeds. One-way ANOVA was performed for significance analysis. Each bar represents three biological replicates ± SD. Different lowercase letters ** indicate significant differences between groups (** *p* < 0.001).

For this study, we first collected data on the germination rate, germination potential, germination index, relative salt injury rate, and salt tolerance indicators of the two *Agropyron* varieties, aiming to explore their performance at the seed stage under sodium chloride treatment. During the seed stage, the two varieties exhibited significant differences under various low-concentration NaCl treatments. Significant differences between *A. desertorum* (Fisch.) Schult and *A. desertorum* (Fisch.) cv. Nordan were mainly seen during the low-NaCl-concentration treatment. At a sodium chloride concentration of 60 mM, the relative germination potential of *A. desertorum* (Schult.) was significantly higher than that of *A. desertorum* cv. Nordan (see Figure 3A). Regarding the relative germination rate, the two varieties exhibited significant differences under varying NaCl concentrations. Specifically, at 30 and 60 mM, *A. desertorum* Schult’s relative germination rates were 20.8% and 28.3% higher than *A. desertorum* Nordan’s, respectively (Figure 3B). At NaCl concentrations of 60–90 mM, *A. desertorum* Schult’s relative germination index was 4.2% and 3.2% higher than *A. desertorum* Nordan’s, respectively (Figure 3C). Overall, in terms of the relative germination rate, germination potential, and germination index, both varieties exhibited similar trends with increasing NaCl concentrations: an initial decrease at 0–30 mM, a subsequent increase at 30–60 mM, followed by another decrease at 60–180 mM. Overall, NaCl treatment exhibited a clear inhibitory effect, as reflected in the declining trends of these indices, which corresponded with the relative salt injury rate (Figure 3D). Analysis of the relative salt injury rate indicated that *A. desertorum* Schult demonstrated better adaptability at lower concentrations, while both varieties experienced increased toxicity with rising NaCl levels. At 30 and 60 mM, the two varieties exhibited highly significant differences in relative salt injury rates, with *A. desertorum* Schult’s rates being 20.8% and 28.3% lower than *A. desertorum* Nordan’s, respectively, indicating that *A. desertorum* Schult possesses greater salt tolerance at the seed stage. Based on these parameters, we analyzed the adaptation range, semi-lethal concentration range, and extreme concentration range of the two varieties under different NaCl levels. The results showed that at the seed stage, their extreme range was the same (<180 mM), as was their semi-lethal range (<90 mM). However, *A. desertorum* Schult’s optimal concentration range (<90 mM) was larger than *A. desertorum* Nordan’s (<30 mM) (Table 1).

Based on their performance at the seed stage, it could be inferred that under low NaCl concentrations, *A. desertorum* (Schult.) may exhibit stronger salt tolerance than *A. desertorum* cv. Nordan. Under high NaCl concentrations, the germination potential, relative germination rate, and relative germination index of *A. desertorum* (Schult.) were slightly higher than those of *A. desertorum* cv. Nordan, while its relative salt injury rate was slightly lower. However, these differences are not significant.

### 3.2. Morphological and Physiological Responses of Seedlings Under NaCl Stress

#### 3.2.1. Morphological Responses of Seedlings to NaCl Stress

Seed-stage NaCl treatment analysis revealed that the majority of differences between the two varieties occurred at lower (30 and 60 mM) salt concentrations. At higher concentrations (90, 120, and 150 mM), the two varieties exhibited comparable responses. Consequently, NaCl concentration gradients of 0, 30, 60, 120, and 180 mM were chosen to assess salt tolerance during the seedling stage. To thoroughly evaluate the growth responses of the two varieties (*A. desertorum* Schult, S; *A. desertorum* Nordan, N) under different NaCl treatments, leaf length, leaf width, and root length were measured at the same developmental stage to assess their salt tolerance phenotypes. The findings for these growth parameters under varying salt concentrations are summarized below.

**Figure 4:**
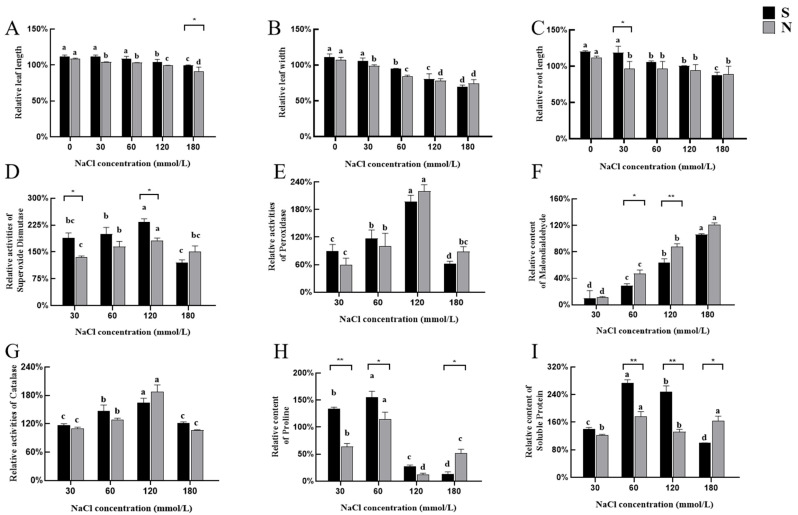
Effects of salt stress on the phenotypic and physiological indicators of two varieties. (**A**–**C**) Leaf length, leaf width and root length of *A. desertorum* Schult and *A. desertorum* Nordan under salt stress. (**D**–**I**) Physiological parameters of S and N under salt stress, including SOD, POD, MDA, CAT, PRO, and SP, respectively, Note: S: *A. desertorum* Schult; N: *A. desertorum* Nordan. One-way ANOVA was performed for significance analysis. Each bar represents three biological replicates ± SD. Different lowercase letters indicate significant differences at *p* < 0.05. * and ** indicate significant differences between groups (* *p* < 0.05; ** *p* < 0.001).

Without NaCl treatment, *A. desertorum* Schult and *A. desertorum* Nordan exhibited leaf length increases of 3.16 cm and 3 cm, respectively, resulting in relative leaf lengths exceeding 100%. Under NaCl treatment, both species showed a general trend of reduced leaf growth as salinity stress increased. At 180 mM NaCl, *A. desertorum* Nordan exhibited a significant decrease in leaf length (*p* < 0.05) (Figure 4A). Regarding leaf width without NaCl treatment, *A. desertorum* Schult and *A. desertorum* Nordan showed average increases of 0.03 cm and 0.04 cm, respectively, compared to the initial measurements. However, as NaCl concentrations increased, leaf width significantly decreased, particularly at 120 mM and 180 mM (*p* < 0.05). This trend was more pronounced in *A. desertorum* Nordan (Figure 4B). In terms of root length, both materials showed a significant reduction at 180 mM (*p* < 0.05). However, when the NaCl concentration was ≤30 mM, the decrease in *A. desertorum* Schult’s root length was relatively gradual (Figure 4C).

#### 3.2.2. Physiological Responses of Seedlings to NaCl Stress

Under salt stress conditions, *A. desertorum* Nordan and *A. desertorum* Schult exhibited significant differences in stress resistance, as evidenced by variations in their relative contents. In terms of oxidative stress defense, the differences between the two varieties of *A. desertorum* were mainly reflected when the concentration of sodium chloride was 60 millimoles per liter and 120 millimoles per liter. Both plants exhibited peak SOD and POD activities under moderate salt stress (120 mM NaCl). Under extreme salt stress (180 mM NaCl), as shown in the figure, *A. desertorum* Nordan’s SOD and POD activities were slightly higher than those of *A. desertorum* Schult.

Meanwhile, except at the low concentration of 30 mM NaCl, where the MDA levels of both species were similar, *A. desertorum* Schult’s MDA content was significantly lower than *A. desertorum* Nordan’s as the salt concentration increased (*p* < 0.05), indicating less membrane damage in *A. desertorum* Schult (Figure 4F). Regarding CAT activity, both species reached peak levels at 120 mM NaCl; however, no significant differences were observed between them overall (Figure 4G). Additionally, at salt concentrations ranging from 30 to 120 mM NaCl, the PRO and SP levels in *A. desertorum* Schult consistently exceeded those in *A. desertorum* Nordan. However, although both species exhibited severe salt-induced damage as the salt concentration increased, at 180 mM, *A. desertorum* Nordan demonstrated slightly stronger osmotic regulation and cell protection abilities (Figure 4H,I). Both materials reached peak levels at 120 mM and showed a significant decline at 180 mM; therefore, the salt-sensitive concentration for seedlings of both materials is considered to be below 180 mM.

### 3.3. A. desertorum Genes Assembled from Transcriptome Data

*A. desertorum* Schult and *A. desertorum* Nordan exhibited significant physiological stress responses, especially at 120 mM NaCl, enabling a more detailed investigation into the molecular pathways and genes underlying their unique salt tolerance mechanisms. This study selected 120 mM NaCl as the optimal stimulus concentration for transcriptomic analysis. Samples of *A. desertorum* Nordan and *A. desertorum* Schult were collected at different time points (0, 6, 24 h), with 6 and 24 h chosen as the primary time points for transcriptomic analysis. By focusing on this concentration, we aimed to uncover the key regulatory networks underlying the adaptation of these species to high-salinity stress to provide valuable insights for improving crop salt tolerance.

Among the 18 samples derived from the two materials, the raw sequencing reads for Nordan ranged from 44,148,800 to 48,409,294. For Schult, the range was from 41,711,320 to 49,162,844. After filtering, the clean reads in Nordan ranged from 42,976,708 to 47,832,372, while those in Schult ranged from 40,451,394 to 47,820,442 (Table S1). For each sample, the Q20 value was above 96.87%, and the Q30 value exceeded 91.17%. Due to the absence of a reference genome, the clean reads from both materials were assembled into transcripts, which were subsequently used as the transcriptome and reference sequence. A total of 188,112 coding genes were identified. The average length of the coding regions was 1400 bp, with the majority (83,708 coding genes) falling within the range of 1000–2000 bp. Additionally, 39,094 coding genes had lengths exceeding 2000 bp (Table S2).

#### 3.3.1. Functional Annotation of All Non-Redundant Unigenes

**Figure 5:**
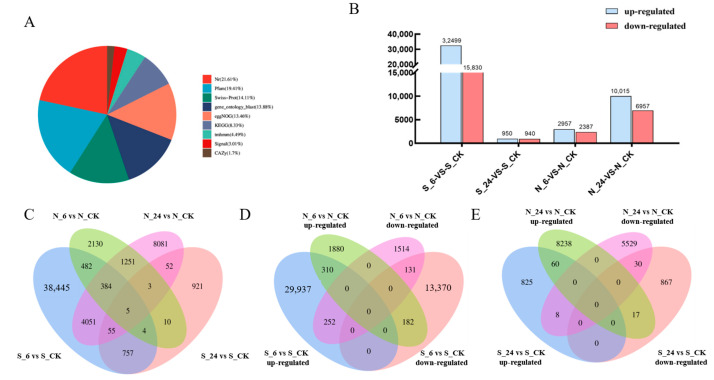
Transcriptome data analysis. (**A**) A comprehensive summary of annotations for all transcripts across various databases was compiled. (**B**) Total number of upregulated and downregulated DEGs in *A. desertorum* Schult and *A. desertorum* Nordan at 6 h and 24 h compared with the control. (**C**) Venn diagram of all DEGs in *A. desertorum* Schult and *A. desertorum* Nordan at 6 h (S_6 and N_6) and 24 h (S_24, N_24). (**D**) Venn diagram of upregulated and downregulated DEGs in *A. desertorum* Schult and *A. desertorum* Nordan at 6 h (S_6, N_6) of salt stress. (**E**) Venn diagram of upregulated and downregulated DEGs in *A. desertorum* Schult and *A. desertorum* Nordan at 24 h (S_24, M_24) of salt stress.

For this study, we used de novo transcriptomic analysis. All of the transcripts obtained from transcriptome sequencing were successfully annotated in various databases, and statistics were compiled (Figure 5A). The results showed that 155,411 transcripts (21.61%) had matches in the Nt database (NCBI nucleotide sequences). Further, 139,562 transcripts (19.41%) had significant matches in the Protein Families Database (Pfam), and 101,454 transcripts (14.11%) had significant matches in the Swiss-Prot database. The number of significant matches was 99,808 (13.88%) in the Gene Ontology (GO) database, 96,761 (13.46%) in the NOG database, and 59,909 (8.33%) in the KEGG database. Building on this foundation, a comparative analysis of DEGs in the two varieties was conducted across various time points, revealing DEG expression patterns at 6 and 24 h (Figure 5B). The number of DEGs in *A. desertorum* Schult at 6 h and 24 h was 48,329 and 1890, respectively, while in *A. desertorum* Nordan, the corresponding numbers were 5344 and 16,972. *A. desertorum* Schult demonstrated an earlier response and self-regulation at 6 h, whereas *A. desertorum* Nordan showed a more concentrated response at 24 h.

#### 3.3.2. Overall Gene Expression Patterns of Two Varieties Under NaCl Stress

To investigate gene expression associated with osmotic stress resistance in two *Agropyron* varieties, we performed a Venn diagram analysis of the differentially expressed genes (DEGs) identified at 6 and 24 h after treatment in *A. desertorum* Schult and *A. desertorum* Nordan (Figure 5C). By comparing the overlapping gene expression between different time points and between the two cultivars, we revealed the expression response characteristics and adaptive mechanisms of these varieties under salt stress. The Venn diagrams (Figure 5D,E) illustrate the changes in DEGs for the two varieties at different time points.

The results demonstrated distinct salt stress responses. At the early 6 h time point, *A. desertorum* Schult exhibited a more active response to NaCl stress than *A. desertorum* Nordan, as evidenced by its uniquely upregulated gene count of 29,937 (62.92%), which was approximately 15.9 times higher than that of *A. desertorum* Nordan, and its uniquely downregulated gene count of 13,370 (28.1%), about 8.9 times higher than that of Nordan. By 24 h, the response in *A. desertorum* Schult markedly decreased; in contrast, *A. desertorum* Nordan showed a stronger response at this time, with a uniquely upregulated DEG count of 8238 (52.9%) that was 9.9 times that of *A. desertorum* Schult, and a uniquely downregulated DEG count of 5529 (25.5%) that was 6.4 times that of *A. desertorum* Schult.

At the same treatment time points, the number of shared DEGs was minimal. At 6 h, only 310 genes (0.65%) were commonly upregulated and 131 genes (0.38%) were commonly downregulated; at 24 h, only 60 genes (0.39%) were upregulated and 30 (0.19%) downregulated. Furthermore, at both 6 and 24 h, the number of genes shared between the two varieties but exhibiting opposing responses was very low, with 182 (0.38%) and 17 (0.11%) genes, respectively.

### 3.4. Functional Classification of DEGs According to GO and KEGG Pathways Under Salt Stress

#### 3.4.1. Gene Ontology Enrichment Analysis of DEGs

GO annotation was performed to elucidate the biological functions of these DEGs and their mechanisms under salt stress. Enrichment analysis of unigenes in the biological process (BP) category (Figure S1) was conducted for the time point of 6 h. *A. desertorum* Schult (S6, S_6h vs. S_ck) and *A. desertorum* Nordan (N6, N_6h vs. N_ck) were enriched in 26 and 21 categories, respectively, while at 24 h, *A. desertorum* Schult (S24, S_24h vs. S_ck) and *A. desertorum* Nordan (N24, N_24h vs. N_ck) were enriched in 24 and 26 categories, respectively. At 6 h, both species responded to stress simultaneously, but their performance was very different. *A. desertorum* Schult primarily displayed self-signaling and hormonal regulation processes in response to external stimuli, including the regulation of signaling, cellular response to endogenous stimuli, regulation of cell communication, cellular response to hormone stimulus, and response to oxygen-containing compounds. In contrast, *A. desertorum* Nordan predominantly activated self-defense and immune mechanisms in response to external stimuli, involving processes such as the regulation of defense response, regulation of immune response, defense response signaling pathway (resistance-gene-dependent), regulation of immune system processes, and positive regulation of immune response. These processes indicate that *A. desertorum* Nordan rapidly activated defense mechanisms, potentially mitigating cell damage caused by salt stress through signal transduction and immune activation. *A. desertorum* Nordan tended to mitigate cellular damage caused by salt stress through pathogen-like defense pathways. Unlike *A. desertorum* Nordan, *A. desertorum* Schult exhibited enrichment in additional biological processes, such as system processes, anatomical structure morphogenesis, tube morphogenesis, cell morphogenesis involved in differentiation, and response to oxygen-containing compounds.

At 24 h, both species retained their distinct BP enrichments observed at 6 h, showing significant enrichment in the “response to salt stress” category. With prolonged stress duration, *A. desertorum* Schult expanded its early rapid response by adopting more complex and multi-level adaptive mechanisms, including cellular component assembly in morphogenesis, tube/cell/tissue development, cell projection morphogenesis, and cellular component morphogenesis. Conversely, at 24 h, *A. desertorum* Nordan further activated its immune and antioxidant defense mechanisms with prolonged treatment, entering a broader phase of defense and adaptive adjustments, including response to oxygen-containing compounds, cell wall macromolecule catabolic processes, and response to hormones.

#### 3.4.2. Kyoto Encyclopedia of Genes and Genomes Pathway Enrichment Analysis of DEGs

**Figure 6:**
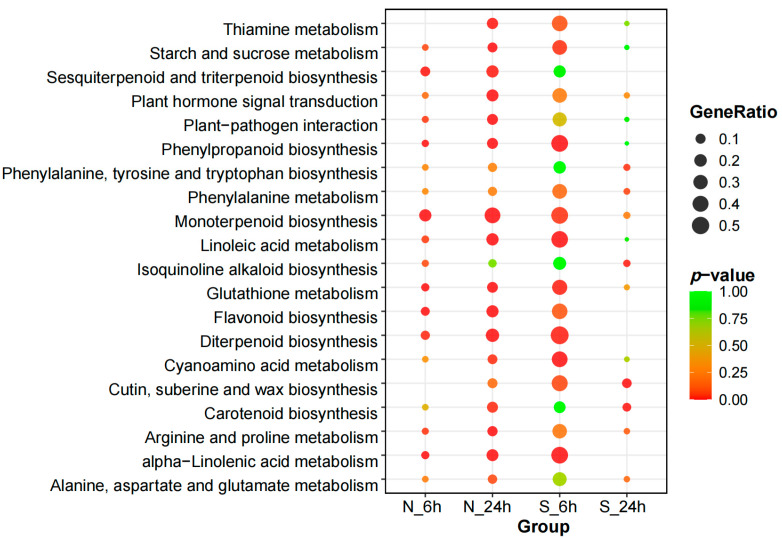
KEGG pathway enrichment analysis of DEGs identified in *A. desertorum* Schult and *A. desertorum* Nordan.

A Kyoto Encyclopedia of Genes and Genomes (KEGG) pathway enrichment analysis was conducted to gain deeper insights into the functional roles of these DEGs from a pathway-specific viewpoint. To visually compare the enrichment of these pathways in the two varieties, a *p*-value threshold of ≤0.05 was set, and the top 20 most enriched pathways in *A. desertorum* Schult and *A. desertorum* Nordan were selected for scatter plot visualization (Figure 6). In analyzing the enriched KEGG pathways of both materials, it became clear that *A. desertorum* Schult and *A. desertorum* Nordan exhibit different strategies for responding to salt stress. After experiencing salt stress, both materials showed responses in the plant–pathogen interaction and plant hormone signal transduction pathways. Additionally, at 6 h, both *A. desertorum* Schult and *A. desertorum* Nordan were significantly enriched in pathways such as phenylpropanoid biosynthesis, monoterpenoid biosynthesis, linoleic acid metabolism, glutathione metabolism, flavonoid biosynthesis, diterpenoid biosynthesis, α-linolenic acid metabolism, and plant hormone signal transduction. However, as indicated by the generation analysis, *A. desertorum* Schult had significantly higher enrichment in these pathways than *A. desertorum* Nordan. At 24 h, the enrichment in *A. desertorum* Nordan showed a general increase, while that in *A. desertorum* Schult showed a decrease, highlighting contrasting patterns. Specifically, in pathways such as thiamine metabolism, starch and sucrose metabolism, plant–pathogen interaction, plant hormone signal transduction, phenylpropanoid biosynthesis, glutathione metabolism, flavonoid biosynthesis, diterpenoid biosynthesis, and cyanoamino acid metabolism, *A. desertorum* Nordan had increased enrichment. On the other hand, *A. desertorum* Schult showed increased enrichment in pathways such as alanine, aspartate, and glutamate metabolism, arginine and proline metabolism, isoquinoline alkaloid biosynthesis, and phenylalanine, tyrosine, and tryptophan biosynthesis. *A. desertorum* Schult adopted proactive structural adjustments and osmotic regulation strategies under salt stress, while *A. desertorum* Nordan tended to maintain cellular stability through metabolic regulation and antioxidant mechanisms. These strategic differences highlight their respective salt tolerance characteristics, providing new insights into the physiological adaptation mechanisms of different varieties under salt stress.

### 3.5. Analysis of DEGs Associated with ROS Scavenging

**Figure 7:**
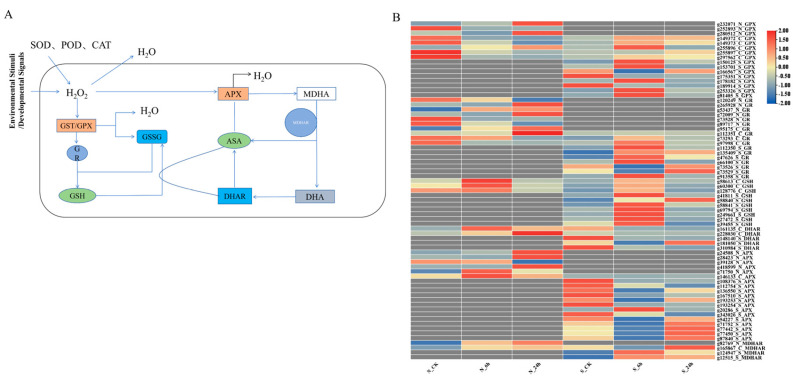
Analysis and comparison of DEGs related to the ASA–GSH pathway in *A. desertorum* Schult and *A. desertorum* Nordan under salt stress. (**A**) Main ASA–GSH pathway under salt stress. (**B**) Heatmap of DEGs related to the ASA–GSH pathway in *A. desertorum* Schult and *A. desertorum* Nordan. For treatments (6 h and 24 h) and control (0 h), upregulated genes are shown in red and downregulated genes are shown in blue.

The production of ROS is a common phenomenon in plants under both normal and stressful conditions. However, under unfavorable or stressful conditions, ROS production exceeds the capacity of the antioxidant defense system. Plants then begin to eliminate excess ROS through enzymatic and non-enzymatic pathways [34]. Among these, the first five components form the AsA-GSH pathway, which plays a crucial role in ROS detoxification in plants (Figure 7A). Focusing on this pathway, we analyzed gene expression levels based on the fragments per kilobase of transcript per million mapped reads (FPKM) values of the relevant genes, creating a gene expression heatmap for different time points (Figure 7B).

Under salt stress, plants produce H_2_O_2_, and the glutathione-related detoxification process begins. First, glutathione peroxidase (GPX) and glutathione S-transferase (GST) use GSH as a substrate for detoxifying ROS and xenobiotic substances, while generating unstable intermediate GSSG. Subsequently, GSSG is regenerated into GSH with the help of NADPH as an electron donor, through the activity of glutathione reductase (GR), maintaining the cellular redox balance. Additionally, DHAR uses GSH to reduce dehydroascorbate (DHA) back to AsA, further assisting in the detoxification of excessive H_2_O_2_ and thus enhancing the plant’s antioxidant capacity [35]. From the heatmap, it can be observed that GSH regulation was very active in both species during non-biotic stress detoxification, especially in the GST pathway, where 163 genes in S and 76 genes in N were identified, with 42 being shared between the two varieties (Figure S2).

Overall, regarding the GSH pathway, *A. desertorum* Schult exhibited significantly more proactive upregulation at 6 h, which gradually weakened over time, and no significant difference in expression was observed later on (Figure 7B). In contrast, *A. desertorum* Nordan showed significant upregulation of GSH genes at 6 h, followed by overall upregulation at 24 h. The maximum log_2_ fold change (log_2_^FC^) values for the GST, GPX, and GR genes in S_6h and N_24h were 5.2 vs. 5.1, 4.21 vs. 3.40, and 4.71 vs. 3.42, respectively. At 6 h, the log_2_^FCmax^ (log_2_^FC^ maximum value) for GSH genes in S and N was 4.09 and 2.08, respectively.

In the AsA pathway, the APX enzyme uses AsA as an electron donor to reduce H_2_O_2_ to water, producing a transient intermediate, MDHA. MDHA is unstable and can be regenerated into AsA through the activity of MDHAR, with a portion spontaneously converting to dehydroascorbate (DHA). Subsequently, DHA is reduced back to AsA by GSH to maintain the cellular redox balance [15] (Figure 7A). From the heatmap of DEG expression levels, it can be seen that the AsA response in both species was concentrated at 24 h. Regarding ROS detoxification in the AsA pathway, the upregulation response in N was more pronounced. The maximum log_2_^FC^ values for APX in S and N at 24 h were 3.1 and 6.7, respectively. The two varieties showed different responses for DHAR. In S, DHAR was significantly downregulated as the stress duration increased, while in N, DHAR was significantly upregulated, with a log_2_^FCmax^ of 1.97 at 24 h. For MDHAR, N exhibited an upregulation trend, with the highest log_2_^FCmax^ at 24 h (0.91), whereas S showed significant upregulation at 6 h, with a log_2_^FCmax^ of 2.71.

## 4. Discussion

The results of this study indicate that under different levels of salt stress, the relative germination rate, relative germination potential, and relative germination index of *A. desertorum* Schult and *A. desertorum* Nordan seeds were affected to varying degrees. This observation is consistent with previous findings for *Cryptotaercia japonica* seeds, showing that as salt concentration increases, the degree of inhibition on seed germination becomes more pronounced. Low concentrations of NaCl have been found to enhance the germination rate of *Sorghum bicolor* seeds, whereas higher concentrations significantly inhibit germination. Studies on 14 plant species with notable stress resistance, including *Dactylis glomerata*, *Vicia sativa*, *Fatsia japonica*, *Medicago sativa*, *Astragalus adsurgens*, and *Lolium perenne*, reported that stress under treatment with 50 mM NaCl promoted germination in some species, whereas higher concentrations (150 and 200 mM NaCl) inhibited germination to varying degrees across all tested species [36]. Under NaCl stress in this study, *A. desertorum* Schult seeds exhibited higher germination rates than *A. desertorum* Nordan seeds at low concentrations of 30 mM and 60 mM. The germination rates of *A. desertorum* Schult and *A. desertorum* Nordan were 20.8% and 28.3% higher at 60 mM than at 30 mM, respectively, suggesting a promotive effect on seed germination at these concentrations. In studies related to the genus Agropyron, it has been found that appropriate salt or drought stress can significantly increase the germination rate of Agropyron plants. When the concentration exceeded 150 mM, the inhibitory effect on seed germination became significant, aligning with the aforementioned findings. In this study, both varieties exhibited similar responses under high NaCl concentrations, a phenomenon also observed in other plants. For instance, in eight soybean (*Glycine max*) varieties subjected to NaCl stress, when NaCl concentrations exceeded 100 mM, parameters such as plant height showed no significant differences among the varieties [37]. Similarly, in an evaluation of salt tolerance among five wheat varieties (Zhoumai 18, Bainong 207, Huayu 198, Aikang 58, and Bainong 69), no significant differences were observed at NaCl concentrations of 150 mM and higher [38].

Based on the evaluation of common stress-resistant plants’ salt tolerance, both *A. desertorum* Schult and *A. desertorum* Nordan exhibited a commendable performance. Notably, *A. desertorum* Schult demonstrated superior salt tolerance at lower NaCl concentrations (≤120 mM), indicating an advantage in adapting to osmotic stress. Consequently, *A. desertorum* Schult may hold greater potential for application in saline regions and under high-osmotic-stress conditions.

ROS play a key role in plant stress signaling. They are generated during aerobic metabolism and include hydrogen peroxide (H_2_O_2_), superoxide anion (O^2−^), nitrogen oxides (e.g., NO^−^), and hydroxyl radicals (·OH). These molecules are highly reactive in plant cells and can attack various macromolecules, such as lipids, proteins, DNA, and RNA, causing oxidative damage [39,40,41]. Under salt stress, the production of ROS affects plant growth and yield [42]. Plants have evolved two primary antioxidant systems: enzymatic systems, including SOD, POD, CAT, GST, GPX, GR, APX, and MDHAR, and non-enzymatic systems, comprising GSH, AsA, α-tocopherol, carotenoids, flavonoids, and phenolics [43]. In this study, after the salt stress treatment, significant differences in the activity of superoxide dismutase (SOD) were observed between the two varieties of *A. desertorum*. The SOD activity of *A. desertorum* (Schult.) was significantly higher than that of *A. desertorum* (Fisch.) Schult. cv. Nordan when the salt concentration was 30 millimoles per liter and 120 millimoles per liter (Figure 4D). Under salt stress at ≤120 mM NaCl, *A. desertorum* Schult exhibited significantly higher Pro and SP contents than *A. desertorum* Nordan, indicating an advantage in osmotic stress adaptation (Figure 4H,I). These findings are consistent with studies on sensitive and tolerant wheat varieties [44], where tolerant varieties, KRL210 [45] and KH65, exhibited higher SP and Pro contents under salt stress [46]. This enhanced osmotic regulation capacity enables *A. desertorum* Schult to adapt more rapidly to salt stress environments, demonstrating superior salt tolerance. MDA, a product of lipid peroxidation, reflects the degree of cell membrane damage in plants under salt stress [47]. A higher MDA content indicates more severe membrane damage [48]. In this study, as salt concentration increased, the MDA content in both species also increased, reaching a maximum at 180 mM. Considering the changes in enzyme activity and osmotic regulatory substance content, 180 mM can be regarded as the salt sensitivity threshold for seedlings of the two varieties evaluated in this study, guiding environmental improvements and applications in saline–alkali land.

In studies comparing the salt-tolerant rice varieties “Nona Bokra” and “C34” with the salt-sensitive cultivar “IR29”, it was observed that the salt-tolerant varieties had a higher number of DEGs than “IR29” [49]. This suggests that salt-tolerant varieties have a greater capacity for transcriptional regulation under salt stress. Similarly, in our study on two varieties, *A. desertorum* Schult displayed a higher number of upregulated and downregulated DEGs in the early stages of stress (6 h). As the duration of stress increased, the number of DEGs in *A. desertorum* Schult decreased, a pattern also observed in *Cynodon dactylon* (L.) Pers., where the number of DEGs increased initially from 1 to 24 h under salt stress before gradually decreasing [50]. This phenomenon may indicate that *A. desertorum* Schult rapidly adjusts its gene expression within the first 6 h to adapt to the stress environment and becomes acclimated to the salt stress by 24 h. In contrast, *A. desertorum* Nordan exhibited an increase in DEGs over time in response to stress. This observation is further supported by GO and KEGG annotations. In the BP analysis, *A. desertorum* Schult’s DEGs at 6 h were enriched in pathways related to stress signal transduction and responses to oxygen-containing compounds (Figure S1). Conversely, *A. desertorum* Nordan showed enrichment in responses to oxygen-containing compounds at 24 h. In the KEGG analysis, the DEGs in both *A. desertorum* Schult and *A. desertorum* Nordan were enriched in pathways such as terpene biosynthesis, glutathione metabolism, and flavonoid biosynthesis, all of which are associated with salt stress (Figure 4). The degree of enrichment in these pathways corresponded to the temporal changes in DEG numbers.

Furthermore, the application of AsA and GSH has been shown to enhance plants’ antioxidant defenses and overall tolerance to abiotic stresses. Thus, AsA and GSH not only scavenge ROS but also help maintain homeostasis in the cytoplasm and other organelles, thereby reducing the oxidative damage induced by various abiotic stresses [51]. In this study, we analyzed the transcriptional regulation patterns of the AsA-GSH antioxidant pathway under salt stress in *A. desertorum* Schult and *A. desertorum* Nordan, revealing distinct antioxidant response strategies between the two. Regarding GSH detoxification, *A. desertorum* Schult displayed a higher number of differentially expressed GST genes. In the GSH pathway, relevant genes in *A. desertorum* Schult showed significant upregulation at 6 h, whereas in *A. desertorum* Nordan, upregulation was prominent at 24 h (Figure S1 and Figure 7). The log_2_^FCmax^ of these genes was higher in *A. desertorum* Schult compared to *A. desertorum* Nordan. In studies on sesame (*Sesamum indicum*) under salt stress, it was observed that the expression of GSH-related genes increased over time [52]. This may explain why *A. desertorum* Nordan exhibited lower redox indices than *A. desertorum* Schult at NaCl concentrations ≤ 120 mM, while the decline in root length was less pronounced at NaCl concentrations > 180 mM.

In this study, we found that under salt stress conditions, *A. desertorum* Nordan exhibited a higher log_2_^FCmax^ for APX upregulation at the same time points than *A. desertorum* Schult. Additionally, DHAR expression was downregulated in *A. desertorum* Schult but upregulated in *A. desertorum* Nordan. These findings suggest that the AsA-GSH pathway plays a crucial role in ROS detoxification in both varieties under salt stress. Given their genetic differences, *A. desertorum* Schult may enhance ROS scavenging primarily through the GSH pathway, while *A. desertorum* Nordan appears to maintain redox balance by bolstering the AsA pathway.

In addition, this study analyzed key redox indicators, including SOD, POD, MDA, CAT, SP, and PRO, and compared the expression of genes associated with stress resistance in the AsA–GSH antioxidant pathway between the two varieties under salt stress. These findings not only provide valuable insights into the mechanisms of salt tolerance but also offer potential strategies for breeding salt- and osmotic-stress-resistant varieties for use in salt-affected and high-osmotic-stress environments.

In addition to redox reactions, other hormone signaling pathways also play important synergistic roles in plants’ response to salt stress. For example, the phytohormone abscisic acid (ABA) controls stomatal closure and ion homeostasis via signaling components, including SnRK2s and ABI5, preventing water loss and ensuring ion balance at the cellular level [53]. The plant hormone jasmonic acid (JA) regulates growth and developmental processes under stress conditions and triggers defense responses [54]. Indole-3-acetic acid (IAA) has also been reported to improve oxidative stress tolerance by enhancing antioxidant enzyme activity and promoting photosynthesis [35]. Determining its subsequent DEG expression patterns is difficult because of the genetic differences between the study materials, their different response mechanisms to these DEGs, and the lack of *A. desertorum* Nordan DEGs that have been sequenced and analyzed beyond 24 h. We hope that future studies will increase the duration of the transcriptomic analysis and expand the focus on the response of pathways of key differentially expressed genes (DEGs) to salinity stress to provide a more comprehensive understanding of the functions and regulatory networks of salt-tolerant genes in these two varieties. This strategy will provide a more exhaustive assessment of their salt tolerance and yield insights that can be used in the development of crop varieties tolerant to saline and high-osmotic-stress environments.

## 5. Conclusions

This study provides a comprehensive comparison of the physiological and molecular responses of *A. desertorum* Schult and *A. desertorum* Nordan to salt stress, from seed germination to seedling growth. Compared with *A. desertorum* Nordan, *A. desertorum* Schult exhibited stronger salt tolerance under salt stress at both the seed germination and seedling stages. The results showed that, at the seed germination stage, *A. desertorum* Schult tolerated a broader range of NaCl concentrations. Low concentrations (30–60 mM) promoted germination in both materials. During the seedling stage, *A. desertorum* Schult better mitigated the accumulation of ROS caused by salt stress, which substantially affects plant growth and development. The different performances of *A. desertorum* Schult and *A. desertorum* Nordan across these stages may be attributed to variations in their ROS scavenging capacities. Transcriptomic analysis of both these wheatgrass species under salt stress showed that DEGs were enriched in pathways related to ROS scavenging and hormone regulation. Within these pathways, *A. desertorum* Schult and *A. desertorum* Nordan exhibited many shared enrichment patterns but differed in the timing and intensity of gene regulation. These integrated findings strongly support the evaluation of *A. desertorum* Schult and *A. desertorum* Nordan for salt tolerance, revealing key regulatory networks and response mechanisms crucial for salt tolerance. They also provide valuable insights for cultivating salt- and osmotic-stress-tolerant crop varieties in saline areas. Furthermore, they offer valuable guidance for breeding strategies aimed at enhancing wheatgrass salt tolerance.

## Figures and Tables

**Table 1:** Salt tolerance indices under different salt concentrations.

Salt Tolerance Index	Concentration/%	
	NaCl	
	S	N
Suitable concentration of salt tolerance	<90 mm	<30 mM
Salt-tolerant half-lethal concentration	<60 mM	<60 mM
Salt tolerance limit concentration	<180 mM	<180 mM

## Data Availability

The raw data for the two Agropyron varieties have been successfully submitted to the NCBI BioProject database under BioProject ID PRJNA1221127. The raw data supporting the findings of this study are publicly available in the NCBI Sequence Read Archive (SRA) under the BioProject accession number PRJNA1221127 and can be accessed at https://www.ncbi.nlm.nih.gov/sra/PRJNA1221127 (accessed on 11 February 2025).

## Supplementary Materials

The following supporting information can be downloaded at: https://www.mdpi.com/article/10.3390/genes16040367/s1. Figure S1: GO classification and statistical analysis of DEGs at different time points in two *Agropyron* varieties.; Figure S2: Heatmap of GST–related DEGs in the ASA–GSH pathway for Schult and Nordan. Table S1: Raw and Clean Reads with Quality Metrics; Table S2: Transcript Assembly Length Distribution.
