# Supplementary material for: Comparative Analysis of Salt Tolerance and Transcriptomics in Two Varieties of Agropyron desertorum at Different Developmental Stages
Source: Genes (Basel). 2025 Mar 22;16(4):367. doi: 10.3390/genes16040367 (PMC12026692; doi:10.3390/genes16040367)
Supplement: Supplementary file 1 [file genes-16-00367-s001.zip › Supplementary Figures S1 and S2.pdf]

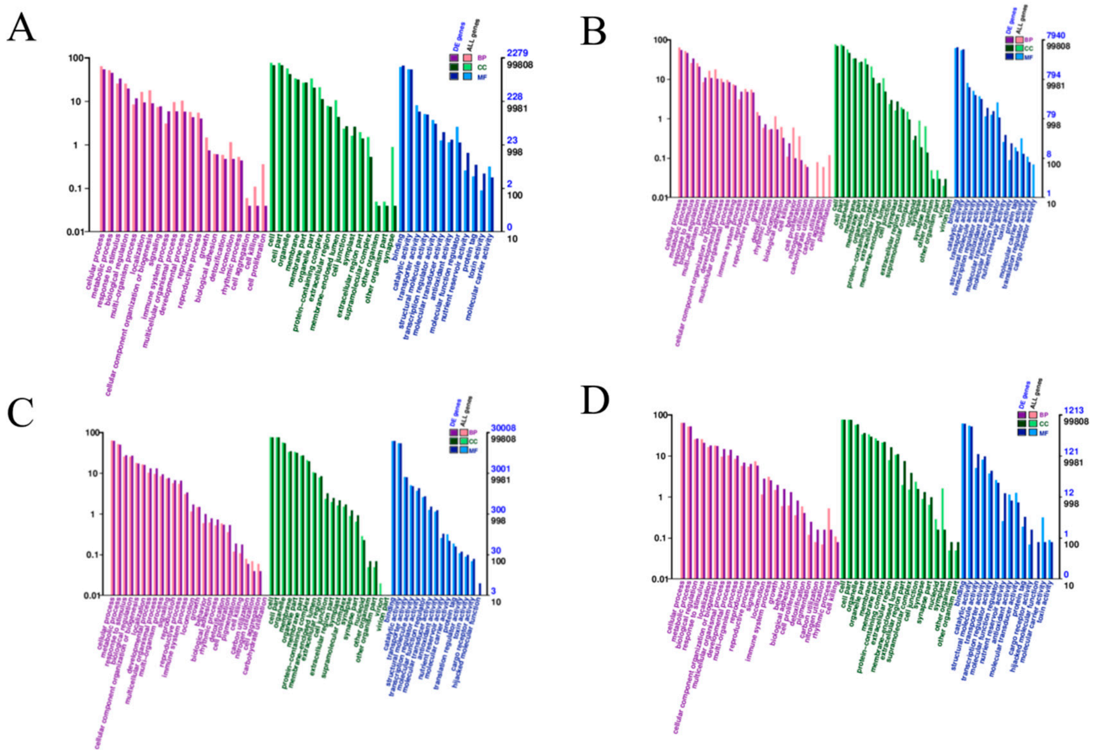

Figure S1. GO classification and statistical analysis of DEGs at different time points in two *Agropyron* varieties. (A−B) Classification and statistical analysis of DEGs in Nordan at 6 h and 24 h. (C−D) Classification and statistical analysis of DEGs in Schult at 6 h and 24 h. The top enriched GO terms in the three main GO categories are shown.

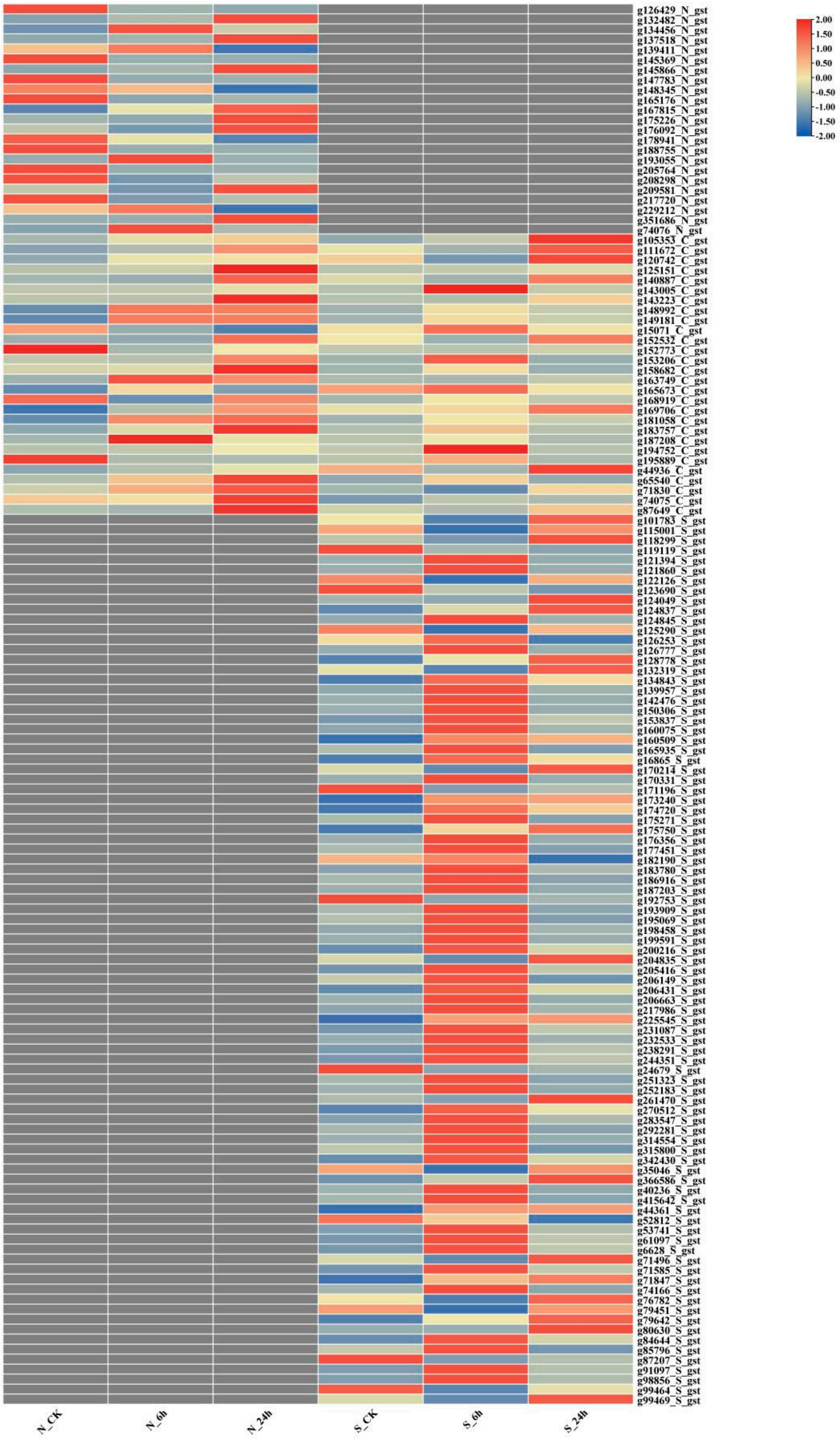

Figure S2. Heatmap of GST-related DEGs in the ASA−GSH pathway for Schult and Nordan. For treatments (6 h and 24 h) and the control (0 h), upregulated genes are shown in red, and downregulated genes are shown in blue.
