# Supplementary figures and images for: Comparative Analysis of Salt Tolerance and Transcriptomics in Two Varieties of Agropyron desertorum at Different Developmental Stages
Source: Genes (Basel). 2025 Mar 22;16(4):367. doi: 10.3390/genes16040367 (PMC12026692; doi:10.3390/genes16040367)

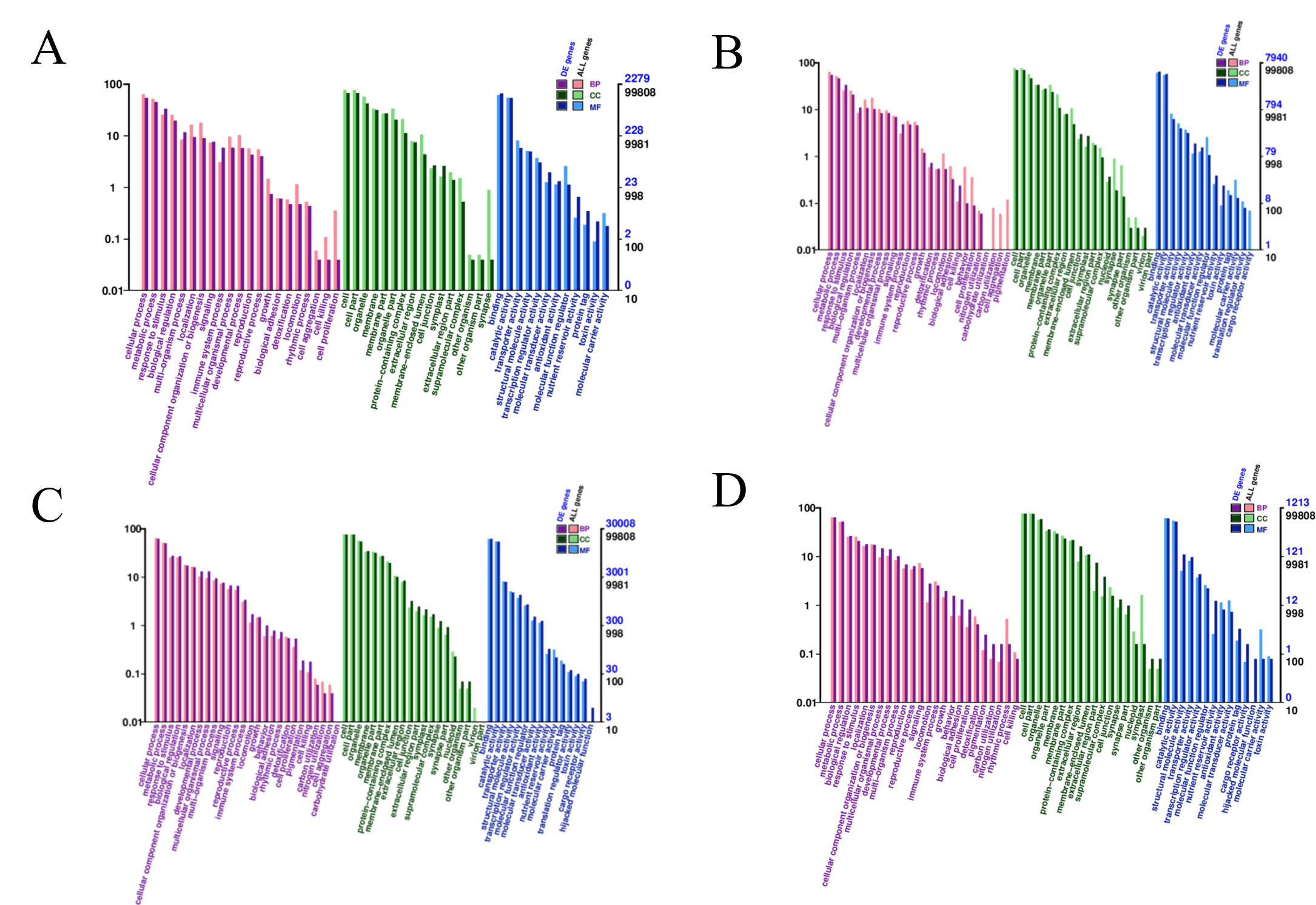

Supplement: Supplementary file 1 [file genes-16-00367-s001.zip › Supplementary Figure S1.png]
